# Aquagenic Wrinkling of the Palms as a Screening Indicator for Cystic Fibrosis Beyond Infancy

**DOI:** 10.7759/cureus.102506

**Published:** 2026-01-28

**Authors:** Alexios Alexopoulos, Dimitrios Ntokos, Despina Briana, Louiza Kontara, Giorgos Chouliaras, Christina Kanaka-Gantenbein, Christina Stefanaki, Lamprini Nasi

**Affiliations:** 1 First Department of Pediatrics, School of Medicine, Agia Sophia Children's Hospital, National and Kapodistrian University of Athens, Athens, GRC; 2 Department of Surveying and Geoinformatics Engineering, School of Engineering, University of West Attica, Egaleo, GRC; 3 Neonatal Intensive Care Unit, Third Department of Pediatrics, School of Medicine, Attikon Hospital, National and Kapodistrian University of Athens, Athens, GRC; 4 Department of Paediatrics, North Middlesex University Hospital, London, GBR; 5 Second Department of Pediatrics, School of Medicine, Aglaia Kyriakou Children's Hospital, National and Kapodistrian University of Athens, Athens, GRC; 6 First Department of Dermatology, School of Medicine, Andreas Sygros Hospital, National and Kapodistrian University of Athens, Athens, GRC

**Keywords:** aquagenic wrinkling of the palms, biw test, cystic fibrosis, diagnostic screening, pediatric dermatology, transepidermal water loss

## Abstract

Aquagenic wrinkling of the palms (AWP) has been associated with cystic fibrosis (CF) and may support screening approaches beyond infancy in low-resource settings. We evaluated the diagnostic accuracy and reproducibility of a standardized brief immersion in water (BIW) test in 100 children with genetically confirmed CF, 50 heterozygous CF transmembrane conductance regulator (CFTR) mutation carriers, and 100 age-matched healthy controls. Both hands were immersed in tap water (22-24°C) for 11 minutes and assessed at 3, 7, and 11 minutes. Transepidermal water loss (TEWL) was measured after immersion using a portable VapoMeter. Early AWP at three minutes occurred in 68/100 (68%) CF patients, 4/50 (8%) CFTR heterozygous carriers, and 0/100 (0%) controls (p < 0.01); at the three-year follow-up, 35/50 (70%) CF patients again showed early wrinkling. Wrinkling with papule formation at seven minutes provided optimal discrimination (94% sensitivity, 98.3% specificity). A TEWL threshold ≥ 203 g/m²/h further differentiated CF from non-CF participants (86% sensitivity, 98% specificity). The BIW test is rapid, reproducible, and feasible in resource-limited settings; thus, a positive seven-minute response should prompt confirmatory testing (e.g., sweat chloride and/or CFTR genotyping).

## Introduction

Aquagenic wrinkling of the palms (AWP) is characterized by rapid, exaggerated palmar wrinkling within minutes of water exposure, distinct from the slower immersion wrinkling seen in healthy individuals. Aside from being a cutaneous curiosity, AWP occurs disproportionately in individuals with cystic fibrosis (CF) and in some CF transmembrane conductance regulator (CFTR) heterozygous carriers, with proposed mechanisms, including altered sweat electrolyte composition, aquaporin dysregulation, and increased transepidermal water permeability [1-13]. Patients may also report swelling, translucent papules, or dysesthesia during immersion [14].

Given these associations, AWP has gained interest as a quick, low-cost screening tool outside newborn programs and beyond infancy, particularly in settings in which sweat chloride testing or CFTR genotyping is limited. Prior reports consistently demonstrated a higher prevalence and faster onset of AWP in individuals with CF than in controls, and time-resolved protocols suggest that early wrinkling and papule formation offer meaningful discriminatory value [1-3,8-10,15-17].

Building on our earlier observational findings in Greek pediatric cohorts [18,19], which demonstrated a strong correlation between AWP and confirmed CF diagnosis, we sought to further evaluate AWP as a clinically actionable screening indicator. In this prospective study, we reassessed 50 children with CF from the original cohort of 100 genetically confirmed patients three years after their initial evaluation. The reproducibility and temporal stability of their brief immersion in water (BIW) responses supported the robustness of this method. Thus, this study aims to refine the diagnostic utility of AWP by applying a structured BIW protocol in a large, genetically confirmed pediatric cohort and incorporating transepidermal water loss (TEWL) measurements to provide objective biophysical validation.

## Materials and methods

Study design and setting

A prospective observational study was carried out at Agia Sophia Children's Hospital in Athens, Greece, a national referral center for CF, from January 2023 to December 2024. The study protocol was approved by the Scientific Council (Institutional Review Board) of the General Children’s Hospital, Agia Sophia, Athens, Greece (approval/protocol number: 5526/07.11.2022), and written informed consent was obtained either from the adult participants or the parents or legal guardians of minors, in accordance with the principles of the Declaration of Helsinki.

**Figure 1:**
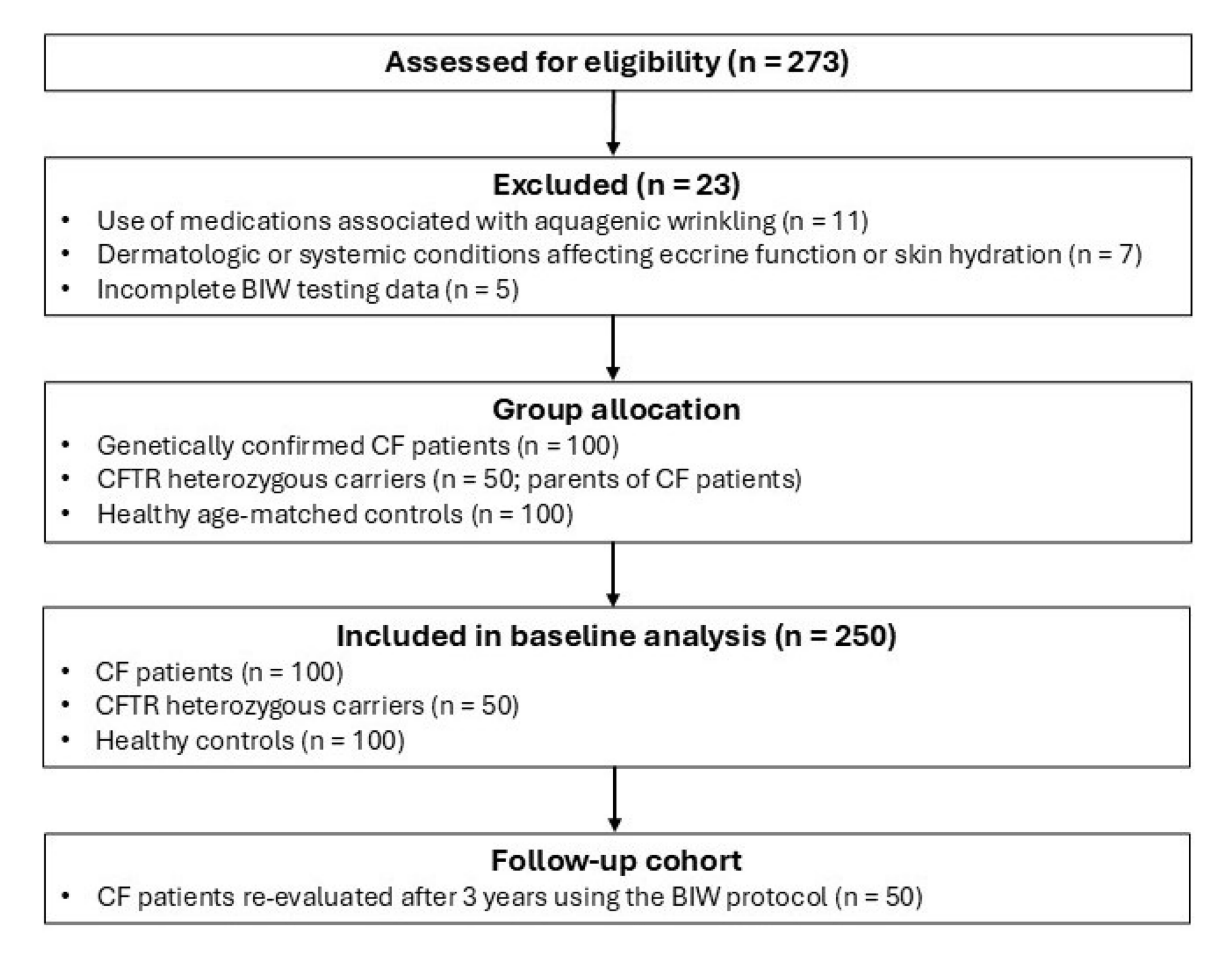
A flow diagram depicting participant recruitment, eligibility assessment, exclusions with reasons, group allocation, and follow-up procedures applied in the study

Three study groups were enrolled at baseline: 100 children with CF (defined by sweat chloride values ≥ 60 mEq/L and the presence of pathogenic CFTR mutations), 50 heterozygous CFTR mutation carriers (biological parents of children with CF), and 100 age-matched healthy controls attending general pediatric clinics without CF or related conditions. The exclusion criteria comprised recent or ongoing use of medications known to be associated with aquagenic wrinkling (including non-steroidal anti-inflammatory drugs (NSAIDs), isotretinoin, gabapentin, and COX-2 inhibitors), as well as any dermatologic or systemic disorder that could affect skin hydration or eccrine gland function. A total of 273 individuals were assessed for eligibility; 23 were excluded (medications associated with AWP, n = 11; dermatologic or systemic conditions affecting eccrine function or hydration, n = 7; incomplete BIW testing data, n = 5), resulting in 250 participants included in the baseline analysis (CF, n = 100; CFTR heterozygous carriers, n = 50; healthy controls, n = 100). To assess reproducibility, 50 children with CF from the original cohort of 100 genetically confirmed patients were re-evaluated three years after their initial assessment using the same BIW protocol. The details regarding participant recruitment, eligibility assessment, group allocation, and follow-up are provided in Figure 1.

BIW procedure

The participants immersed both hands in tap water (22-24°C) for 11 minutes. Palmar surfaces were evaluated at 3, 7, and 11 minutes by a pediatric dermatologist. The following features were documented: visible wrinkling (≥ 30% of the palm), papules, edema, and subjective sensations (pruritus or pain). AWP was defined as coarse or deep wrinkling involving ≥ 30% of the palm, with or without secondary findings.

TEWL assessment

Epidermal barrier function was assessed three minutes after immersion using a Vapometer® (Delfin Technologies, Finland). Three consecutive readings were obtained from the central palm of the dominant hand, averaged, and expressed as g/m²/h.

Statistical analysis

Statistical analyses were performed using Statistical Product and Service Solutions (SPSS, version 26; IBM SPSS Statistics for Windows, Armonk, NY). Continuous variables are presented as mean ± standard deviation or as median with interquartile range (IQR), while categorical variables are summarized as absolute numbers and percentages. Comparisons between groups were conducted using a one-way analysis of variance (ANOVA) with Bonferroni correction or the Kruskal-Wallis test, as appropriate, while categorical data were analyzed using Fisher's exact test. Diagnostic performance was quantified in terms of sensitivity, specificity, positive predictive values (PPVs), negative predictive values (NPVs), and likelihood ratios. Predictive models were also constructed for assumed pre-test CF probabilities of 1%, 10%, and 25% to explore clinical applicability.

Diagnostic accuracy metrics were calculated using standard definitions. Sensitivity was defined as the proportion of true-positive cases correctly identified by the BIW test, while specificity represented the proportion of true-negative cases correctly classified. PPVs and NPVs were calculated based on the assumed a priori disease probabilities. The corresponding formulas are as follows:

$\text{Sensitivity (\%)} = \frac{\mathrm{TP}}{\mathrm{TP} + \mathrm{FN}} \times 100$

$\text{Specificity (\%)} = \frac{\mathrm{TN}}{\mathrm{TN} + \mathrm{FP}} \times 100$

$\text{PPV (\%)} = \frac{\mathrm{TP}}{\mathrm{TP} + \mathrm{FP}} \times 100$

$\text{NPV (\%)} = \frac{\mathrm{TN}}{\mathrm{TN} + \mathrm{FN}} \times 100$

## Results

Participant characteristics and baseline findings

The study cohort comprised 250 participants: 100 children with genetically confirmed CF, 50 CFTR heterozygous carriers, and 100 healthy age-matched controls. The mean age was 10.4 ± 4.0 years in the CF group and 10.5 ± 4.0 years in the controls, while the CFTR heterozygous carriers were older (35.9 ± 6.1 years). Overall age differences across groups were statistically significant (p < 0.001), with pairwise analyses indicating significant contrasts between carriers and both pediatric groups (p < 0.001).

Sex distribution did not differ significantly between the groups (p = 0.076). Nevertheless, male participants were more frequent among CFTR heterozygous carriers (35/50; 70%) than among children with CF (51/100; 51%) or controls (54/100; 54%). Females accounted for 110 of 250 individuals (44%), corresponding to 49% in the CF group, 30% among CFTR carriers, and 46% in the control group.

**Figure 2:**
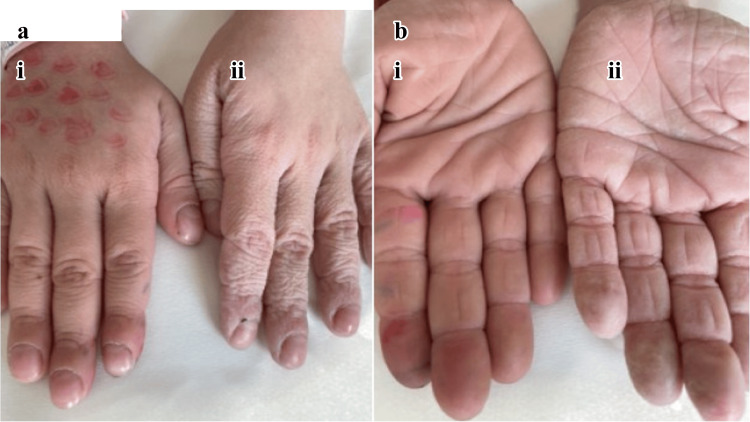
Aquagenic wrinkling of the palms during the BIW test Representative examples of aquagenic wrinkling of the palms during the brief immersion in the water (BIW) test. (a) Child with cystic fibrosis: (i) baseline (0 min), (ii) after seven minutes of water immersion. (b) Healthy control: (i) baseline (0 min), (ii) after seven minutes of water immersion. Time points are reported in the legend and are not displayed on the images.

Among the CF participants, the mean age at diagnosis was 1.1 ± 2.2 years (IQR: 0.2-0.9 years). At the time of the initial BIW testing (Figure 2), the mean disease duration was 9.3 ± 4.0 years, reflecting early diagnosis and sustained clinical follow-up. At re-evaluation, 50 of the original 100 CF patients (mean age: 13.1 ± 4.0 years) underwent repeat BIW testing approximately three years after their initial assessment.

**Figure 3:**
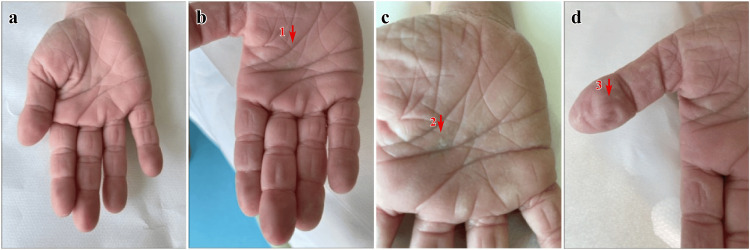
Sequential palmar changes during the BIW test Sequential clinical manifestations observed during the brief immersion in the water (BIW) test. (a) Baseline palmar appearance before immersion. (b) Hyperwrinkling evident after three minutes of water exposure. (c) Marked wrinkling with papule formation after seven minutes. (d) Edema with accentuated cutaneous changes after 11 minutes. Red arrows denote the locations of wrinkling (1), papules (2), and edema (3). Time points are specified in the legend and are not indicated directly on the images.

Cutaneous response profiles differed markedly among the three initial groups. As shown in Table 1, the first CF column displays the findings of the original 100 CF children, while the adjacent CF column presents the results of the 50 children reassessed three years later using the same BIW protocol. Palmar wrinkling, papules, and edema were significantly more prevalent in CF patients than in CFTR heterozygous carriers and control participants at all time points of the initial assessment. By contrast, pruritus and pain at three minutes and seven minutes did not reach statistical significance. Figure 3 presents the sequential palmar changes during the BIW test.

**Table 1:** Cutaneous findings during the BIW test Comparative evaluation of cutaneous findings (edema, papules, wrinkling, pruritus, and pain) among children with cystic fibrosis (CF) at baseline and follow-up, cystic fibrosis transmembrane conductance regulator (CFTR) heterozygous carriers, and healthy controls following the brief immersion in the water (BIW) test. Data are presented as numbers (percentage). Statistical comparisons refer exclusively to baseline group comparisons.

Time (minutes)	Clinical finding	CF Initial (n = 100)	CF Follow-up (n = 50)	CFTR heterozygous carriers (n = 50)	Controls	p-value
3	Edema	22 (22%)	10 (20%)	0 (0%)	0 (0%)	<0.01*
	Papules	46 (46%)	24 (49%)	1 (2%)	0 (0%)	<0.01*
	Wrinkles	68 (68%)	35 (70%)	4 (8%)	0 (0%)	<0.01*
	Pruritus	5 (5%)	4 (8%)	1 (2%)	0 (0%)	0.07*
	Pain	1 (1%)	2 (3%)	0 (0%)	0 (0%)	0.99*
7	Edema	56 (56%)	27 (54%)	1 (2%)	0 (0%)	<0.01*
	Papules	94 (94%)	46 (92%)	9 (18%)	1 (1%)	<0.01*
	Wrinkles	100 (100%)	50 (100%)	16 (32%)	7 (7%)	<0.01*
	Pruritus	14 (14%)	7 (14%)	1 (2%)	0 (0%)	<0.01*
	Pain	3 (3%)	2 (4%)	0 (0%)	0 (0%)	0.23*
11	Edema	79 (79%)	38 (76%)	8 (16%)	7 (7%)	<0.01*
	Papules	100 (100%)	49 (98%)	26 (52%)	19 (19%)	<0.01*
	Wrinkles	100 (100%)	50 (100%)	49 (98%)	84 (84%)	<0.01*
	Pruritus	41 (41%)	21 (42%)	2 (4%)	1 (1%)	<0.01*
	Pain	10 (10%)	5 (10%)	0 (0%)	0 (0%)	<0.01*

The p-values shown in Table 1 correspond exclusively to the comparisons between CF, CFTR heterozygous carriers, and control groups during the initial assessment and do not apply to the follow-up subgroup. The follow-up CF data are presented descriptively to illustrate the reproducibility and temporal stability of the BIW responses.

TEWL measurements confirmed the pattern of greatest severity in CF patients (259.0 ± 43.4 g/m²/h; median 268.5 (IQR 249.0-290.0)), followed by CFTR heterozygous carriers (183.5 ± 27.2; median: 184.0 (175.0-195.0)) and controls (158.3 ± 30.8; median: 154.0 (140.5-181.0)). The one-way ANOVA showed significant intergroup differences (p < 0.01), with Bonferroni-corrected post-hoc tests confirming all pairwise contrasts (p < 0.001). In the reassessment, the 50 CF children demonstrated a comparable profile, with 70% early wrinkling and a slightly lower mean TEWL value (237.5 g/m²/h; median: 245.0 (230.0-260.0)), thus supporting the reproducibility and stability of the BIW test.

Diagnostic performance in CF identification

All diagnostic performance analyses referred to the initial assessments of the three study groups. As summarized in Table 2, palmar wrinkling and papule formation at seven minutes demonstrated the strongest discriminatory capacity for CF identification across low (1%), moderate (10%), and high (25%) pre-test probability scenarios. A TEWL cut-off of ≥ 203 g/m²/h optimized diagnostic performance, yielding 86% sensitivity, 98% specificity, and 92% overall accuracy in distinguishing CF from non-CF participants. These metrics remained stable across all probability models.

**Table 2:** Diagnostic performance of the BIW test for cystic fibrosis detection Diagnostic performance of the brief immersion in the water (BIW) test outcomes for distinguishing cystic fibrosis (CF) from non-CF participants (cystic fibrosis transmembrane conductance regulator (CFTR) heterozygous carriers and healthy controls) at 3, 7, and 11 minutes. Sensitivity and specificity are reported as percentages. Positive predictive values (PPVs) and negative predictive values (NPVs) were calculated assuming a priori CF probabilities of 1%, 10%, and 25%.

Time (minutes)	CF vs CFTR heterozygous carriers/controls	Sensitivity (%)	Specificity (%)	A priori probability 1%		A priori probability 10%		A priori probability 25%	
				PPV (%)	NPV (%)	PPV (%)	NPV (%)	PPV (%)	NPV (%)
3	Edema	22.0	100.0	100.0	99.2	100.0	92.0	100.0	79.4
	Papules	46.0	99.9	85.3	99.5	98.5	94.3	99.5	84.7
	Wrinkle	68.0	99.7	68.2	99.7	95.9	96.6	98.6	90.3
	Pruritus	5.0	99.9	38.7	99.0	87.4	90.4	99.5	85.7
	Pain	1.0	100.0	100.0	99.0	100.0	90.1	100.0	75.2
7	Edema	56.0	99.9	87.6	99.6	98.7	95.3	99.6	87.2
	Papules	94.0	98.3	36.1	99.9	86.1	99.3	94.9	98.0
	Wrinkle	100.0	92.0	11.2	100.0	58.1	100.0	80.6	100.0
	Pruritus	14.0	99.9	63.9	99.1	95.1	91.2	98.3	77.7
	Pain	3.0	100.0	100.0	99.0	100.0	92.8	100.0	75.6
11	Edema	79.0	92.6	9.8	99.8	54.3	97.5	78.2	93.0
	Papules	100.0	79.7	4.7	100.0	35.5	100.0	62.1	100.0
	Wrinkle	100.0	15.4	1.2	100.0	11.6	100.0	28.3	100.0
	Pruritus	41.0	98.9	27.0	99.4	80.3	93.8	92.4	83.4
	Pain	10.0	100.0	100.0	99.1	100.0	90.9	100.0	76.9

Screening limitations for CFTR heterozygous carriers

As shown in Table 3, diagnostic accuracy was substantially lower when distinguishing CF heterozygous carriers from controls. Assuming a 4% population prevalence of CFTR heterozygous carriers, a TEWL threshold of ≥ 171 g/m²/h achieved a 72% correct classification rate. Although some BIW outcomes demonstrated high PPVs (up to 100%), NPVs (≈ 96%) were modest, indicating limited reliability for carrier exclusion. Thus, although the BIW test is valuable for CF screening, its utility for generalized carrier detection should be interpreted cautiously, particularly in low-prevalence populations.

**Table 3:** Diagnostic performance of the BIW test for CFTR heterozygous carrier identification Diagnostic performance of the brief immersion in the water (BIW) test outcomes for distinguishing cystic fibrosis transmembrane conductance regulator (CFTR) heterozygous carriers from healthy controls at 3, 7, and 11 minutes. Sensitivity, specificity, positive predictive values (PPVs), and negative predictive values (NPVs) are reported as percentages. “N.a.” indicates instances in which PPVs could not be calculated because of the absence of positive cases.

Time (minutes)	BIW outcome	Sensitivity (%)	Specificity (%)	PPV (%)	NPV (%)
3	Edema	0.0	100	n.a.	96.0
	Papules	2.0	100	100	96.1
	Wrinkle	8.0	100	100	96.3
	Pruritus	2.0	100	100	96.1
	Pain	0.0	100	n.a.	96.1
7	Edema	2.0	100	100	96.1
	Papules	18.0	99.0	42.8	96.7
	Wrinkle	32.0	93.0	16.0	97.0
	Pruritus	2.0	100	100	96.1
	Pain	0.0	100	n.a.	96.0
11	Edema	16.0	93.0	8.7	96.4
	Papules	52.0	81.0	10.2	97.6
	Wrinkle	98.0	16.0	4.6	99.5
	Pruritus	4.0	99.0	14.3	96.1
	Pain	0.0	100	n.a.	96.0

## Discussion

AWP is a distinctive but polymorphic dermatologic phenomenon observed in three principal contexts: (i) idiopathic presentations, often in adolescent females, that typically remit spontaneously [4-7]; (ii) secondary forms associated with CF or CFTR mutations [1,2,8-11,14]; and (iii) drug-induced cases linked to NSAIDs, COX-2 inhibitors, gabapentin, isotretinoin, and certain antibiotics, such as tobramycin and clarithromycin [6,7]. Its pathophysiological substrate in CF likely reflects altered sweat electrolyte composition, aquaporin dysregulation, and impaired epidermal barrier function [12,13]. Although AWP lacks specificity when observed in isolation, its diagnostic value is strengthened through standardized protocols, such as the BIW test.

This study is among the largest to evaluate AWP under a structured immersion protocol in a demographically stratified pediatric CF cohort. The reassessment of CF patients after three years, demonstrating comparable BIW responses and timing profiles, confirms the reproducibility and temporal stability of the method. Consistent with previous reports [1,15,16], we observed a reproducible, time-dependent pattern, with wrinkling and papule formation at seven minutes, providing optimal diagnostic performance and significant intergroup differences evident at three minutes (Figure 2). These findings are aligned with previous data: Arkin et al. reported AWP in 84% of CF patients versus 0% of controls [1], while Tolland et al. and Garçon-Michel et al. demonstrated similar prevalence patterns [16,17]. Gild et al. provided further granularity, documenting wrinkling onset at 3, 7, and 11 minutes in CF, carriers, and controls, respectively [10]. Similarly, Singh et al. showed that 75% of children with CF wrinkled within three minutes when sweat chloride levels exceeded 60 mEq/L [15].

Our protocol also incorporated an objective biophysical dimension, with a TEWL threshold ≥ 203 g/m²/h yielding 86% sensitivity and 98% specificity for CF detection. These findings are consistent with previous Greek pediatric studies [18,19]. The agreement between visual and biophysical markers reinforces the diagnostic validity of AWP in CF.

Conversely, the BIW test demonstrated a limited capacity to distinguish CFTR heterozygous carriers from controls. Although certain BIW outcomes exhibited high PPVs, the NPVs remained modest, mirroring prior case-control data [10] and highlighting the limited utility of BIW for generalized carrier screening.

Emerging evidence continues to expand the understanding of AWP expression. Reports of AWP in CF patients receiving CFTR modulators [20] and during the COVID-19 pandemic in adolescents [21] underscore its dynamic response to genetic and environmental factors. Case reports, such as that of Aparício Martins et al. [22], and Manoh et al.'s demonstration of BIW utility in malnourished infants [23] further support its relevance as a simple and accessible screening approach.

Newborn screening remains central to CF diagnosis in high-income countries, typically combining immunoreactive trypsinogen with DNA analysis and confirmatory sweat chloride testing (≥ 60 mEq/L) [24]. However, interpretive challenges associated with CFTR variants of variable penetrance [25] have renewed interest in phenotype-based adjuncts, such as the BIW test.

Advances in artificial intelligence (AI) offer additional prospects for AWP assessment. Yang et al. proposed AI-supported image analysis and photographic standardization [26], which may improve reproducibility, may enable teledermatology applications, and are particularly valuable in remote or resource-limited settings. Such approaches are aligned with the European Cystic Fibrosis Society (ECFS) recommendations aimed at promoting equitable access to CF care [27].

The BIW test remains a low-cost, non-invasive, repeatable, and feasible tool during a single outpatient visit. With minimal equipment (i.e., tap water, a timer, and visual inspection), it can be effectively implemented in primary care, school, or community settings lacking molecular diagnostics. Evaluating wrinkling, papules, swelling, itching, and pain at 3, 7, and 11 minutes provides temporal resolution and interpretive consistency. AWP is considered positive when more than 30% of the palm surface exhibits wrinkling (Figure 3), in accordance with a recent expert consensus [26].

Although not a definitive diagnostic test, the BIW protocol serves as a rapid triage tool that can guide confirmatory evaluation, particularly in symptomatic individuals beyond infancy. Nonetheless, several limitations should be acknowledged. The absence of examiner blinding may have introduced observer bias; thus, future studies should incorporate blinded assessments or inter-rater reliability analysis. Age differences between pediatric CF patients and adult CFTR heterozygous carriers could confound TEWL comparisons, suggesting the need for age-matched cohorts. Environmental factors, such as water temperature, humidity, and diurnal variation, were not stringently controlled and could have influenced TEWL measurements. Multicenter studies conducted under standardized environmental conditions could help establish normative TEWL ranges and enhance external validity.

## Conclusions

This study reaffirms the diagnostic value of the BIW test as a simple, reproducible, and cost-effective screening tool for cystic fibrosis beyond infancy. Although not intended to replace confirmatory methods, such as sweat chloride testing or CFTR genotyping, the BIW test fills an important diagnostic gap in resource-limited or decentralized settings by facilitating the early identification of high-risk or symptomatic individuals. By combining clinical observation with TEWL measurement using a portable Vapometer, this approach enhances diagnostic precision and reproducibility. With further validation and potential digital adaptation, the BIW protocol may serve as a first-line dermatologic triage tool that can expand global access to timely CF diagnosis and reduce diagnostic inequities.
